# The Rieske Iron-Sulfur Protein: Import and Assembly into the Cytochrome *bc*_1_ Complex of Yeast Mitochondria

**DOI:** 10.1155/2011/363941

**Published:** 2011-05-18

**Authors:** Laura Conte, Vincenzo Zara

**Affiliations:** Dipartimento di Scienze e Tecnologie Biologiche ed Ambientali, Università del Salento, Via Prov.le Lecce-Monteroni, 73100 Lecce, Italy

## Abstract

The Rieske iron-sulfur protein, one of the catalytic subunits of the cytochrome *bc*_1_ complex, is involved in electron transfer at the level of the inner membrane of yeast mitochondria. The Rieske iron-sulfur protein is encoded by nuclear DNA and, after being synthesized in the cytosol, is imported into mitochondria with the help of a cleavable N-terminal presequence. The imported protein, besides incorporating the 2Fe-2S cluster, also interacts with other catalytic and non-catalytic subunits of the cytochrome *bc*_1_ complex, thereby assembling into the mature and functional respiratory complex. In this paper, we summarize the most recent findings on the import and assembly of the Rieske iron-sulfur protein into *Saccharomyces cerevisiae* mitochondria, also discussing a possible role of this protein both in the dimerization of the cytochrome *bc*_1_ complex and in the interaction of this homodimer with other complexes of the mitochondrial respiratory chain.

## 1. Introduction

The Rieske iron-sulfur protein (Rip1p or ISP) is one of the catalytic subunits of the cytochrome *bc*_1_ complex (also known as complex III of the mitochondrial respiratory chain). This respiratory enzyme assembles as a mature and functional homodimer in the inner membrane of mitochondria; besides ISP, it incorporates two further catalytic subunits, cytochrome *b* and cytochrome *c*_1_, and seven noncatalytic subunits (core protein 1 and core protein 2, Qcr6p, Qcr7p, Qcr8p, Qcr9p, Qcr10p). These last subunits are also named “supernumerary” because they are absent in some bacterial equivalents of cytochrome *bc*_1_ complex [1, 2]. ISP is essential for the activity of this complex and for the respiratory growth of *Saccharomyces cerevisiae *mitochondria [3–5]. The gene *RIP1* is conserved across both prokaryotes and eukaryotes [2], and its human homolog is UQCRFS1. A comparison of the amino acid sequence has revealed a high degree of similarity, especially in the C-terminal region of the protein. Functionally, ISP strictly cooperates with cytochrome *b* and cytochrome *c*_1_ in the electron transfer catalyzed by the mitochondrial cytochrome *bc*_1_ complex. In doing so, ISP requires the presence of a 2Fe-2S cluster, which is bound to the movable C-terminal domain of the protein located in the mitochondrial intermembrane space. This intermembrane space domain is connected by a short amino acid linker to the hydrophobic N-terminal *α*-helix, which is inserted into the inner mitochondrial membrane. The studies carried out over the last decades on the import and assembly of ISP into mitochondria have revealed a well-defined pathway of interactions of this protein with several *bc*_1_ complex subunits. In addition, the topology of ISP in the large homodimeric cytochrome *bc*_1_ complex structure is quite characteristic; indeed, while the transmembrane domain of ISP strictly interacts with the *bc*_1_ subunits of one monomer, the intermembrane space domain of the same protein strictly interacts with the *bc*_1_ subunits of the other monomer. Therefore, the implications of ISP biogenesis and assembly appear intriguing. It is indeed possible that ISP, besides representing an obligatory component for the redox activity of the *bc*_1_ complex, may also play a role in building up the mature and functional complex III in the mitochondrial membranes.

## 2. Structural Characteristics of ISP in the Cytochrome *bc*_1_ Complex

**Figure 1:**
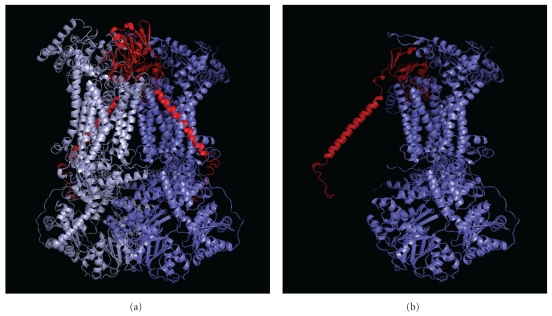
Topology of ISP in the yeast cytochrome *bc*_1_ complex. (a) The arrangement of the Rieske protein in the *bc*_1_ complex homodimer. The subunits in the cytochrome *bc*_1_ dimer are depicted as ribbons, with subunits in one monomer colored grey and subunits in the other monomer colored cyan. ISPs of the two monomers are colored red. (b) Panel B shows the spatial arrangement of ISP (red) in one cytochrome *bc*_1_ monomer (cyan), in order to highlight its transdimeric structure.

The resolution of the crystal structure of the cytochrome *bc*_1_ complex isolated from beef [6, 7], chicken [8], and yeast [9] mitochondria has revealed a dimeric structural arrangement of this complex. In the crystal structure, the two copies of ISP are intertwined and oriented in a *trans* manner with respect to each monomer (Figure 1). Each molecule of ISP is localized on the outer surface of the inner mitochondrial membrane and consists of three separate domains: (i) a membrane-spanning *α*-helix domain at its N-terminus; (ii) a soluble C-terminal extramembranous domain containing the bulk of the protein and the iron-sulfur cluster; (iii) a short segment of 7 to 9 amino acids connecting the extrinsic domain to the membrane-spanning domain [7–9].

The structures of the N-terminal domains of yeast and bovine/chicken Rieske proteins are different, and this difference coincides with a low sequence homology [9]. The amino terminus of yeast ISP is relatively enriched in polar amino acids [3]. There is a stretch of hydrophobic amino acids distal to the N-terminus, beginning at alanine 55; this hydrophobic sequence and the polar amino acids anchor ISP to the *bc*_1_ complex [2]. More in detail, the first ten residues of the N-terminal polypeptide of yeast ISP form one strand of a *β* sheet to which cytochrome *c*_1_, Qcr8p, and core protein 1 contribute [9]. This secondary element is also present in the bovine subunit [7]. In the mammalian crystal structure, the transmembrane *α*-helix of ISP interacts with the transmembrane helices of Qcr9p and Qcr10p [7]. Despite the low sequence homology and because of the similar overall structure of the yeast compared with the bovine and chicken complexes, the authors assumed that the location of the transmembrane helix may be very similar in the complexes [8, 9]. 

The structure of the soluble extramembranous domain of yeast ISP [9] is highly similar to the homologous domain of the bovine complex [7]. This extrinsic domain (residues 93–215) is a flat spherical module containing three layers of antiparallel *β* sheets and forms a functional unit with cytochrome *b* and cytochrome *c*_1_ of the second monomer. The iron-sulfur cluster is coordinated by Cys159, His161, Cys178, and His181 with Cys164 and Cys180 forming a disulfide bridge, which stabilizes the cluster [9, 10]. Failure to insert the iron-sulfur cluster impairs assembly of ISP into the *bc*_1_ complex, as evidenced by the susceptibility of this catalytic subunit to protease degradation [11]. Eliminating the disulfide bridge did not significantly impair the stability of the cluster but indirectly damaged the ubiquinol oxidation site [12]. The destruction of the Rieske iron-sulfur cluster by hematoporphyrin-promoted photoinactivation resulted in a proton-permeable *bc*_1_ complex [13]. Another study confirmed that the elimination of the iron-sulfur cluster in mutant *bc*_1_ complexes opened up an otherwise closed proton channel within this respiratory complex [14]. Therefore, it was speculated that in the normal catalytic cycle of the *bc*_1_ complex, the iron-sulfur cluster may function as a proton-exiting gate regulating the controlled, vectorial extrusion of protons across the *bc*_1_ complex [14]. 

One of the striking features of the cytochrome *bc*_1_ complex structures was the finding that the catalytic domain of ISP, which carries the iron-sulfur cluster, is connected to the transmembrane anchor by a flexible linker region, also called tether domain. In fact, despite the changes in the position of the iron-sulfur cluster, the structures of the extrinsic and the membrane-spanning domains remain unchanged in all of the crystal structures, suggesting that the movement of the iron-sulfur cluster results from the rotation of the flexible linker, which allows the catalytic domain to move during electron transfer between two positions, proximal to cytochrome *b* and to cytochrome *c*_1_. This motion of the catalytic domain of ISP is essential to shuttle reducing equivalents from ubiquinol to the heme group of cytochrome *c*_1_ [7, 8, 15, 16]. The tether domain, a highly conserved region of ISP, shows the sequence TADVLAMA in yeast (residues 85–92). The presence of three highly conserved alanine residues in this region suggests that these small amino acid residues may provide the needed flexibility for the proposed stretching of the “tether” [17]. Mutations that limit the flexibility of the linker region caused a significant decrease in the activity of cytochrome *bc*_1_ complex [18–22]. These results demonstrated that the flexibility of this region is essential for electron transfer activity, consistent with the movement of ISP. Other modifications, such as changes of the length of the linker region, impair the interaction of ubiquinol with cytochrome *bc*_1_ complex [19, 23, 24]. These mutations probably alter the distance between ISP and cytochrome *b* in such a way that the cleft between these two subunits, which is the ubiquinol oxidase site, does not properly accommodate ubiquinol. Thus, it appears that the tether domain can tune the flexibility, the mobility, and the positioning of ISP to allow the finest movement of this protein between cytochrome *b* and cytochrome *c*_1_ during catalysis. However, recent structural and biochemical evidence demonstrated that also cytochrome *b* controls the motion of ISP through its affinity for the ISP extrinsic domain, and this intricate mechanism explains the high fidelity of the electron transfer in the cytochrome *bc*_1_ complex [25–28].

## 3. Import and Maturation of ISP into Yeast Mitochondria

Like the majority of mitochondrial proteins, ISP is encoded by a nuclear gene, translated on cytosolic ribosomes as a precursor protein, and then imported into mitochondria with the help of an N-terminal presequence [29, 30]. The import of ISP and the two-step processing of the precursor protein to its mature size was first characterized in *Neurospora crassa* mitochondria [31]. As in the case of import of other mitochondrial proteins, the entry of ISP into the inner mitochondrial membrane required a membrane potential across the lipid bilayer. After import into energized mitochondria, the protein became resistant to externally added proteases because of its integration into the inner mitochondrial membrane. In *Saccharomyces cerevisiae*, the precursor protein is made up of 215 amino acids and includes an N-terminal presequence of 30 amino acids. This presequence is cleaved off in two steps inside mitochondria, thus generating a mature ISP of 185 amino acids [32–34]. At first, the matrix processing peptidase (MPP) removes a 22-amino acid peptide from the N-terminal of the ISP precursor protein (p-ISP) producing the intermediate form of this protein (i-ISP). The mitochondrial intermediate peptidase (MIP) then removes an octapeptide from i-ISP to generate the mature length iron-sulfur protein (m-ISP), which contains 185 amino acids. On the contrary, in mammals the presequence is cleaved off in a single step and retained inside the cytochrome *bc*_1_ complex [35]. The biological function of the intermediate octapeptide and the reason for the two-steps processing occurring only in distinct ISP precursors belonging to certain organisms, such as *Saccharomyces cerevisiae*, are not fully understood. However, it has been demonstrated that the two-step processing of the ISP precursors is not essential for the import of the protein into mitochondria and its assembly into the cytochrome *bc*_1_ complex; in fact, mutated forms of ISP that are processed to mature size in a single step are properly and functionally assembled [36].

It was also controversial whether i-ISP was first incorporated into the *bc*_1_ complex and then cleaved [37–39] or whether only m-ISP could be assembled into the *bc*_1_ complex [33]. Site-directed mutagenesis experiments demonstrated that the second processing step takes place after i-ISP has been assembled into the yeast mitochondrial *bc*_1_ complex and that the iron-sulfur cluster is inserted into the apoprotein before MIP cleaves off the second part of the presequence [40]. Interestingly, it was also found that i-ISP was not stably inserted into the complex unless cytochrome *c*_1_ was processed to its mature size. In fact, the block of cytochrome *c*_1_ maturation found in a yeast mutant strain lacking Qcr6p also impaired *in situ* maturation of ISP [41]. These findings suggest that the import and assembly of the various subunits into the cytochrome *bc*_1_ complex occur at different rates in an ordered manner. 

In this context, it is important to highlight that also the assembly of iron-sulfur cluster is a complex process involving multiple highly conserved components [42, 43]. In eukaryotes, mitochondria perform a central role in iron-sulfur cluster maturation because they harbor the so-called ISC (iron-sulfur cluster) assembly machinery. In general, maturation of mitochondrial iron-sulfur proteins, such as ISP, involves two major steps [44, 45]. First, an iron-sulfur cluster intermediate is transiently assembled on a scaffold protein (ISCU in humans or Isu1 or Isu2 in yeast) and, subsequently, transferred to the target apoproteins. The sulfur originates from cysteine via the activity of the cysteine desulfurase Nfs1, which is coordinated with the essential accessory protein Isd11. Although the source of sulfur for iron-sulfur cluster formation is clear, the pathway of iron to Isu1 is less defined; however, frataxin (Yfh1 in yeast), a small acidic mitochondrial protein, has been suggested to serve as the iron donor of the reaction through formation of a complex with Isu1, Nfs1, and Isd11 [46–49]. In the second step, the cluster is released from Isu1, transferred to recipient apoproteins, and assembled into the apoprotein by coordination with specific amino acid residues. These processes need the assistance of specific ISC assembly components: the mitochondrial monothiol glutaredoxin Grx5, the dedicated chaperone of the Hsp70 family termed Ssq1, the DnaJ-like cochaperone Jac1, and the nucleotide exchange factor Mge1 [50–55]. However, further studies are necessary to characterize the sequence of events of the early steps of *de novo* iron-sulfur cluster biogenesis.

## 4. Assembly of ISP into the Cytochrome *bc*_1_ Complex

Due to the complexity of the molecular events involved, a discussion on ISP assembly cannot be made without considering the assembly of the cytochrome *bc*_1_ complex in which ISP inserts and operates [56]. Many experiments have been carried out over the last decades on the molecular mechanisms and steps involved in the assembly of the various protein subunits into the cytochrome *bc*_1_ complex. Since the beginning, it appeared plausible that the assembly of these subunits occurred through distinct steps involving the preliminary formation of small *bc*_1_ subcomplexes followed by their ordered interaction in building up the mature and functional *bc*_1_ complex. These hypotheses were formulated indirectly by assaying the steady-state levels of the remaining subunits in the mitochondrial membranes of yeast strains in which genes for one or more *bc*_1_ subunits had been deleted [57–60]. Different experimental approaches, consisting in the solubilization of the wild-type *bc*_1_ complex with detergents and salts, led to the same conclusions, that is the possible existence of specific *bc*_1_ subcomplexes [57, 61]. 

On the basis of these investigations, the existence of at least three distinct *bc*_1_ subcomplexes was proposed: the cytochrome *b*-Qcr7p-Qcr8p subcomplex, the core protein 1-core protein 2 subcomplex, and the cytochrome *c*_1_-Qr6p-Qcr9p subcomplex. It is interesting to note that ISP was not included in any of these subcomplexes, thereby leading to the proposal that this catalytic subunit could be one of the last proteins inserted into the growing cytochrome *bc*_1_ complex. Such a conclusion was also reached analyzing the *bc*_1_ subunit composition of a yeast mutant strain in which the gene encoding ISP had been deleted (ΔISP) [57, 59]. Comparable results were obtained some years later with a ΔQCR10 strain, in which the gene encoding the supernumerary subunit Qcr10p had been deleted [62]. In both yeast mutant strains (ΔISP and ΔQCR10), all the *bc*_1_ subunits were found, with the obvious exception of ISP and Qcr10p. These findings suggest that all the other subunits are able to assemble in a protease-resistant form of the *bc*_1_ complex even in the absence of ISP or Qcr10p. It is in fact known that the imported, but non-assembled, *bc*_1_ subunits are susceptible of protein degradation inside yeast mitochondria [58, 63]. The two strains ΔISP and ΔQCR10, however, showed a striking functional difference because the first was respiratory-deficient whereas the second was respiratory-competent. This is due to the different role played by the two proteins in the mature cytochrome *bc*_1_ complex; in fact, whereas ISP is involved in the electron transport activity catalyzed by the *bc*_1_ complex, Qcr10p is a supernumerary subunit apparently devoid of any role in the respiratory activity. Interestingly, some authors suggested that Qcr10p could be added after the processing of i-ISP to m-ISP [40]. In addition, in the crystal structure of the bovine enzyme, the homologous supernumerary subunit interacts with ISP and is peripherally located [7, 8]. Taken together these data are in favour of a possible interaction of ISP and Qcr10p during the last steps of *bc*_1_ complex assembly. 

An interaction between ISP and the supernumerary subunit Qcr9p was also suggested. In fact, a structural and functional defect of ISP was found in a mutant yeast strain (ΔQCR9) in which the gene encoding Qcr9p had been deleted [64]. In the ΔQCR9 strain the conformation of ISP was altered in such a way that the apoprotein was not able to insert the iron-sulfur cluster appropriately [64]. In addition, in this yeast mutant strain ISP resulted more sensitive to endogenous proteolytic degradation [60, 64].

It is important to underline that the existence of these *bc*_1_ subcomplexes has been postulated only indirectly on the basis of the analysis of the subunit composition of yeast mutant strains in which single genes or pairs of genes encoding distinct *bc*_1_ subunits had been deleted. More recently, a physical interaction between the *bc*_1_ subunits has been directly demonstrated by a different experimental approach. This latter consisted in the analysis of the subunit composition of the *bc*_1_ complex with a non-denaturing electrophoretic technique, the blue-native polyacrylamide gel electrophoresis (BN-PAGE) [65], followed by immunoblotting with antibodies directed against all the *bc*_1_ protein subunits. This approach led to the identification of a new *bc*_1_ subcomplex of approximately 66 kDa, made up of the two subunits ISP and Qcr9p [66]. This was the first demonstration of a direct physical interaction between ISP and another protein subunit of the cytochrome *bc*_1_ complex. In the past, on the basis of indirect evidence [58, 67, 68], an interaction of Qcr9p with cytochrome *c*_1_, instead of ISP, was proposed. A ternary subcomplex, made up of cytochrome *c*_1_, Qcr6p, and Qcr9p, was for a long time retained to be an important intermediate during the assembly of the cytochrome *bc*_1_ complex [57–60]. However, the recent approach of BN-PAGE failed to reveal this *bc*_1_ subcomplex. Interestingly, the 66 kDa subcomplex, derived from the association of ISP and Qcr9p, was only found in yeast mutant strains strongly defective in *bc*_1_ complex assembly, such as the ΔCOR1, ΔCOR2, and ΔCYT1 strains [66]. Indeed, in all these strains only some low MW *bc*_1_ subcomplexes, catalytically inactive, were repeatedly found [66]. These findings suggest that ISP can be isolated in a definite subcomplex with Qcr9p only when the incorporation of these two proteins into the nascent *bc*_1_ complex is inhibited. On the other hand, these results do not guarantee that the 66 kDa subcomplex represents a real *bc*_1_ complex assembly intermediate. They only suggest that ISP is able, in certain conditions, to strongly interact with the supernumerary subunit Qcr9p. Furthermore, no data are currently available on the possible presence in this 66 kDa subcomplex of other protein components, such as specific chaperone proteins which may have a role in *bc*_1_ complex maturation.

When comparing these data with those obtained with the crystal structure of the yeast cytochrome *bc*_1_ complex [9] some new implications interestingly emerged. In fact, the transmembrane region of ISP was found in the vicinity of the corresponding transmembrane region of Qcr9p. More precisely, 14 amino acid residues of the hydrophobic *α*-helix of ISP were found within 4 Å of distance from 12 residues belonging to the hydrophobic *α*-helix of Qcr9p. The particularity is that this interaction between ISP and Qcr9p is established in one *bc*_1_ monomer, whereas the catalytic function of ISP is exerted within the other *bc*_1_ monomer. In fact, it is at the level of the other *bc*_1_ monomer that the functional interaction between ISP and cytochrome *c*_1_ occurs. These results are therefore in agreement with the transdimer structure of ISP within the mature homodimeric *bc*_1_ complex (Figure 1).

## 5. Insertion of ISP into a Core Structure of the Cytochrome *bc*_1_ Complex

Further information on the ISP biogenesis was obtained by investigating the molecular characteristics of a recently identified *bc*_1_ subcomplex of about 500 kDa [69]. This subcomplex was identified in three yeast mutant strains, in which the genes encoding ISP, Qcr9p, or Bcs1p were individually deleted [69]. The minimal composition of this 500 kDa *bc*_1_ subcomplex was found in the ΔQCR9 mutant strain and consisted of cytochrome *b*, cytochrome *c*_1_, core protein 1, core protein 2, Qcr6p, Qcr7p, and Qcr8p. Interestingly, this subcomplex also contained Bcs1p, a chaperone involved in cytochrome *bc*_1_ assembly [70, 71]. The 500 kDa *bc*_1_ subcomplex was proteolytically stable inside yeast mitochondria, was repeatedly found in several yeast mutant strains, and for all these characteristics was therefore named “*bc*_1_ core structure.” In addition, this *bc*_1_ subcomplex was particularly helpful because it allowed a detailed characterization of the last steps of *bc*_1_ assembly, including the precise events leading to ISP integration into the respiratory complex. It was indeed discovered that the binding of Qcr9p preceded the binding of ISP and that the binding of this catalytic subunit was required for the integration of Qcr10p into the mature and functional *bc*_1_ complex [69]. Furthermore, besides Qcr9p, also the presence of the chaperone protein Bcs1p in the 500 kDa subcomplex was absolutely required for the integration of the catalytic subunit ISP [69]. This finding is in agreement with previous results showing the importance of Bcs1p in the biogenesis of ISP and therefore of the entire *bc*_1_ complex. In fact, it has been proposed that the ATP-dependent chaperone Bcs1p maintains the preassembled *bc*_1_ complex in a competent state capable of binding ISP and Qcr10p [71]. It is, however, interesting to find that the presence of only one of these two subunits (Qcr9p or Bcs1p) does not substitute for the other [69]. This means that both proteins, the supernumerary subunit Qcr9p and the chaperone protein Bcs1p, are required for the insertion of ISP into the *bc*_1_ core structure. It is also possible that different structural and/or functional elements of these proteins are absolutely required for the assembly of ISP into the *bc*_1_ complex. 

The existence of this stable *bc*_1_ subcomplex in various yeast mutant strains strongly supports the hypothesis that it may represent a real intermediate during the process of assembly of the homodimeric cytochrome *bc*_1_ complex. However, the possibility that this 500 kDa *bc*_1_ subcomplex may instead represent an incorrectly assembled intermediate or a degradation product characteristic of the yeast mutant strains examined cannot be totally excluded. As a consequence, the data on the pathway followed by ISP during its incorporation into the *bc*_1_ complex might be incorrect or inconclusive. In order to overcome this possible pitfall, Bcs1p was overexpressed in the yeast mutant strain ΔBCS1 containing the 500 kDa *bc*_1_ subcomplex [72], in order to investigate the possibility of a recovery of the mature cytochrome *bc*_1_ complex. Interestingly, the overexpressed Bcs1p was able to reconstruct the functional homodimeric cytochrome *bc*_1_ complex in a time-dependent fashion [72]. This was not the case when some mutant Bcs1p's, instead of the wild type Bcs1p, were overexpressed in the ΔBCS1 yeast strain. These mutant forms of Bcs1p were not able to recover the functional *bc*_1_ complex, even when the coexpression of ISP was also carried out [72]. These findings therefore validated the previous results concerning the sequence of molecular events involved in the assembly of ISP into the *bc*_1_ core structure, which, in its turn, represents a *bona fide* assembly intermediate of the cytochrome *bc*_1_ complex.

## 6. Is ISP Involved in the Dimerization of the *bc*_1_ Complex and/or in the Formation of the Respiratory Chain Supercomplexes?

**Figure 2:**
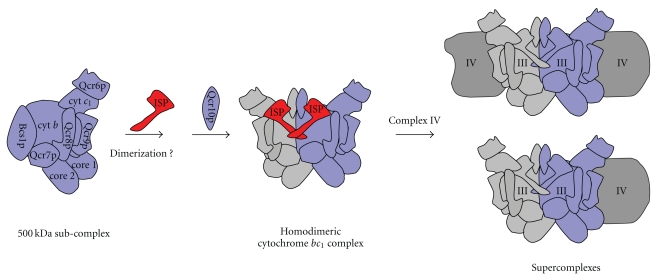
Schematic model depicting the last steps of assembly of the yeast cytochrome *bc*_1_ complex. The 500 kDa *bc*_1_ subcomplex, which also includes the chaperone protein Bcs1p, sequentially binds ISP and, finally, Qcr10p, in a process which leads to the formation of the homodimeric *bc*_1_ complex. In the final step, supercomplexes assembly involves the sequential addition of cytochrome *c* oxidase complex (complex IV) to homodimeric cytochrome *bc*_1_ complex (complex III).

Although the subunit composition of the *bc*_1_ core structure has been carefully analyzed [69], no data are currently available on its aggregation state that could be either monomeric or dimeric. In fact, the molecular mass of 500 kDa could be due to a monomeric state of the immature *bc*_1_ complex associated to still unknown assembly factors or, alternatively, to a dimeric state of the immature complex plus a single copy of the chaperone Bcs1p. Previous findings have shown that the sole integration of ISP into the *bc*_1_ core structure determines a shift in the molecular mass of this complex from 500 to 670 kDa [69]. This change in the size of the complex is too large to be explained by the inclusion of just ISP and Qcr10p (which, as stated before, is added after ISP binding) into the 500 kDa core structure. On the other hand, the molecular shift observed is too small if a *bc*_1_ dimerization occurs at this stage as a consequence of ISP binding. In addition, very little is known on the nature and the role played by the *bc*_1_ assembly factors in the course of these molecular events. We cannot therefore exclude that ISP integration into the *bc*_1_ core structure triggers a general rearrangement of the *bc*_1_ complex and of the putative bound assembly factors eventually leading to the dimerization of the functional complex in the inner mitochondrial membrane (Figure 2). 

The cytochrome *bc*_1_ complex is not only functionally but also structurally associated to other complexes of the mitochondrial respiratory chain. In fact, it has been clearly demonstrated that supercomplexes between cytochrome *bc*_1_ and cytochrome *c* oxidase exist in the inner membrane of yeast and mammalian mitochondria [66, 73, 74]. In particular, two supercomplexes of about 1000 and 850 kDa have been identified in the yeast mitochondrial membranes solubilized with the non-denaturing detergent digitonin and analyzed by BN-PAGE [66, 73]. It has been proposed that these two supercomplexes correspond to the homodimeric cytochrome *bc*_1_ complex plus one copy (850 kDa) or two copies (1000 kDa) of the monomeric cytochrome *c* oxidase complex. The role of distinct subunits of the *bc*_1_ or the oxidase complexes in gluing together these respiratory complexes is currently under investigation. 

On the one hand, it has been proposed that the formation of the 1000 kDa supercomplex requires the presence of the functionally assembled cytochrome *bc*_1_ and cytochrome *c* oxidase complexes [73]. In the absence of ISP the formation of this supercomplex was hindered as a consequence of the lack of an assembled cytochrome *bc*_1_ complex. On the other hand, in the same ΔISP yeast mutant strain the authors found some respiratory complexes, smaller than the supercomplex, yet containing protein subunits belonging to both the *bc*_1_ and the *c* oxidase complexes. This suggests that in the absence of ISP an association between the *bc*_1_ and the oxidase complexes, even if incomplete and non-productive, does occur. 

On the contrary, subsequent experiments revealed that in the absence of ISP no binding between the *bc*_1_ core structure and the mature *c* oxidase complex was possible [69]. In fact, the 500 kDa *bc*_1_ subcomplex found in the ΔISP yeast mutant strain did not contain the oxidase complex which, instead, migrated in its monomeric form in the molecular mass region of about 230 kDa. This suggests that the integration of ISP into the *bc*_1_ complex is an essential prerequisite for the subsequent formation of the supercomplexes (Figure 2). Therefore, these results open up new avenues of investigation on the possible role of ISP in gluing together the respiratory complexes in the higher structures of the supercomplexes. The difficulty of all these studies is due to the necessity of identifying genuine assembly intermediates and not casual protein aggregates that could lead to wrong results and misleading interpretations.

## 7. Concluding Remarks

ISP has a fundamental role in the electron transport activity catalyzed by the cytochrome *bc*_1_ complex because it represents the primary acceptor of electrons deriving from the oxidation of ubiquinol. The catalytic domain of this protein subsequently cooperates with the corresponding domain of cytochrome *c*_1_ in the intermembrane space, where the respiratory flux of electrons occurs. Many studies have therefore been carried out on the functional properties of ISP along with the molecular characterization of the flexibility of its catalytic domain. 

However, also the molecular events involved in the biogenesis of ISP have gained some attention, especially in these last years. The reason for this is due to several factors, first of all to the complicated relationships (structural, spatial, and temporal) existing between the assembly of ISP and that of other protein subunits into the *bc*_1_ complex, which must acquire a homodimeric structure (20 subunits in total) in the inner membrane of *Saccharomyces cerevisiae* mitochondria. Secondly, ISP is the unique subunit of the *bc*_1_ complex showing a transdimeric topology in the functional complex. This structural property could be in some way responsible for *bc*_1_ complex dimerization or it can be at least related to the process of dimerization. The addition of ISP to the growing *bc*_1_ complex may trigger a complex series of events leading to a general rearrangement of the complex and of the bound assembly factors. As discussed before, some hints already exist about a pivotal role of ISP during *bc*_1_ assembly, even if a significant amount of work is still necessary in order to better explore this intriguing possibility. Finally, preliminary data indicate that ISP is also required for the formation of the respiratory supercomplexes in the inner mitochondrial membrane. Whether this effect is indirectly due to the lack of a mature *bc*_1_ complex (in the absence of ISP) or to a direct interaction of ISP with a putative partner subunit of the oxidase complex is a further matter of future investigations.

## 8. Abbreviations

ISP and Rip1p, iron-sulfur protein and Rieske iron-sulfur protein, respectively, indicate the same protein subunit belonging to the yeast cytochrome *bc*_1_ complex; Qcr6p, Qcr7p, Qcr8p, Qcr9p, and Qcr10p, subunits 6, 7, 8, 9, and 10 of the yeast cytochrome *bc*_1_ complex, respectively.
